# Full blood count and haemozoin-containing leukocytes in children with malaria: diagnostic value and association with disease severity

**DOI:** 10.1186/1475-2875-7-109

**Published:** 2008-06-12

**Authors:** Thomas Hänscheid, Matthias Längin, Bertrand Lell, Marc Pötschke, Sunny Oyakhirome, Peter G Kremsner, Martin P Grobusch

**Affiliations:** 1 Medical Research Unit, Albert Schweitzer Hospital, Lambaréné, Gabon; 2 Institute of Molecular Medicine, Lisbon Medical College, Lisbon, Portugal; 3 Department of Parasitology, Institute of Tropical Medicine, University of Tübingen, Tübingen, Germany; 4 Infectious Diseases Unit, Infectious Diseases Unit, Division of Clinical Microbiology and Infectious Diseases, National Health Laboratory Service and School of Pathology, Faculty of Health Sciences, University of the Witwatersrand, Johannesburg 2193, 7 York Road, Parktown, South Africa

## Abstract

**Background:**

Diligent and correct laboratory diagnosis and up-front identification of risk factors for progression to severe disease are the basis for optimal management of malaria.

**Methods:**

Febrile children presenting to the Medical Research Unit at the Albert Schweitzer Hospital (HAS) in Lambaréné, Gabon, were assessed for malaria. Giemsa-stained thick films for qualitative and quantitative diagnosis and enumeration of malaria pigment, or haemozoin (Hz)-containing leukocytes (PCL) were performed, and full blood counts (FBC) were generated with a Cell Dyn 3000^® ^instrument.

**Results:**

Compared to standard light microscopy of Giemsa-stained thick films, diagnosis by platelet count only, by malaria pigment-containing monocytes (PCM) only, or by pigment-containing granulocytes (PCN) only yielded sensitivities/specificities of 92%/93%; 96%/96%; and 85%/96%, respectively. The platelet count was significantly lower in children with malaria compared to those without (p < 0.001), and values showed little overlap between groups. Compared to microscopy, scatter flow cytometry as applied in the Cell-Dyn 3000^® ^instrument detected significantly more patients with PCL (p < 0.01). Both PCM and PCN numbers were higher in severe versus non-severe malaria yet reached statistical significance only for PCN (p < 0.0001; PCM: p = 0.14). Of note was the presence of another, so far ill-defined pigment-containing group of phagocytic cells, identified by laser-flow cytometry as lymphocyte-like gated events, and predominantly found in children with malaria-associated anaemia.

**Conclusion:**

In the age group examined in the Lambaréné area, platelets are an excellent adjuvant tool to diagnose malaria. Pigment-containing leukocytes (PCL) are more readily detected by automated scatter flow cytometry than by microscopy. Automated Hz detection by an instrument as used here is a reliable diagnostic tool and correlates with disease severity. However, clinical usefulness as a prognostic tool is limited due to an overlap of PCL numbers recorded in severe versus non-severe malaria. However, this is possibly because of the instrument detection algorithm was not geared towards this task, and data lost during processing; and thus adjusting the instrument's algorithm may allow to establish a meaningful cut-off value.

## Background

Malaria is known to cause several changes in full blood count (FBC) parameters, of which the most prominent are anaemia and thrombocytopaenia [1]. However, in most studies results are often obtained using manual methods, such as haematocrit and manual white blood cell (WBC) differentials, with inherent limitations. For example, the imprecision of manual counts is well known [2], and assuming that only 100 cells are observed and 5% of cells are found, the 95% confidence interval ranges from 1–12%. However, counting 10,000 cells reduces the limit to 4.6–5.4% [3]. Therefore, modern FBC analysers give highly accurate and precise results, and this within 30 – 60 seconds. Unfortunately, their complexity and cost mostly preclude their use in remote malaria-endemic areas.

Haemozoin (Hz), the end product of the detoxification of haem, is phagozytosed by monocytes and granulocytes. Some studies have reported a link between Hz and dyserythopoiesis and anaemia [4-6]. Using microscopic enumeration of Hz-containing leukocytes (PCL), others have found a strong correlation between these cells and severity of malaria [7-9]. However, as has been pointed out before [10], most of these studies suffer from two significant limitations: (i) the relative paucity of PCL and the rather low number of total leukocytes observed, thus causing a high statistical imprecision of microscopically determined counts; (ii) the microscopical counting of PCL is very time-consuming and subjective. Yet, another aspect of PCL is that their detection may be a very useful tool to diagnose malaria [11]. In this context, it is of interest that one FBC analyser series (Cell-Dyn^®^, Abbott, Santa Clara, California) allows the automated detection of Hz-containing cells. The instrument has been shown to be useful in the diagnosis of malaria [12-15]. One such instrument (Cell-Dyn 3000^®^) has been installed in a remote malaria-endemic area in Central Africa (Lambaréné, Gabon). The objective of this study was to investigate the haematological parameters in children with malaria as well as the importance and potential usefulness of the automated detection of PCL.

## Methods

The study took place at the HAS, Lambaréné, Gabon, in 2003 and 2004. The area is mainly tropical rainforest with holoendemic malaria [16,17]. Blood samples were analysed from children presenting for malaria diagnosis and enrolled into the IPTi-SP trial [18] and other studies for which ethical clearance was obtained from the Ethics Committee of the HAS. Full blood count (FBC) results from children up to 12 years were included. On presentation, demographic data and disease severity, following the WHO 2000 severity criteria [19] were recorded. Blood was collected and anticoagulated with EDTA. Giemsa-stained thick smears were prepared and examined for malaria according to the 'Lambaréné method' [20]. In all children with malaria, pigment-containing monocytes (PCM) and granulocytes (PCN) were counted in a Giemsa-stained thick film, counting a total of 100 monocytes and 200 granulocytes, respectively. The anticoagulated blood was analysed within one hour after collection using a Cell-Dyn 3000^® ^(CD3000) instrument (Abbott, Santa Clara, California).

**Figure 1:**
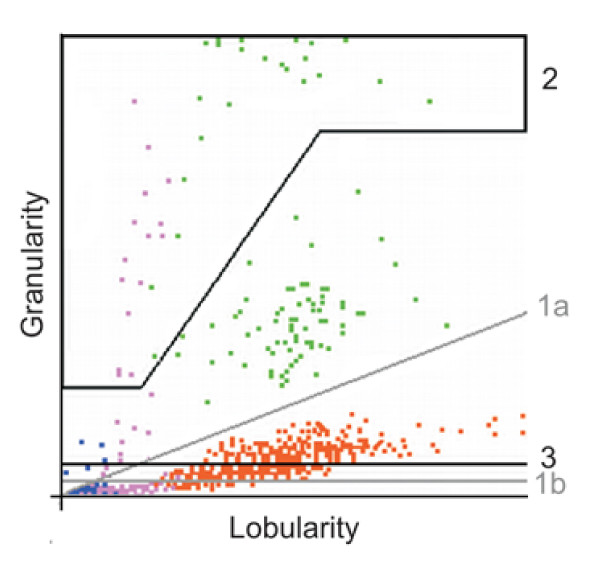
**Granularity/lobularity plot from the Cell-Dyn 3000^®^**. The plot shows the 90° side scatter (lobularity) on the x-axis and the 90° depolarizing scatter on the y-axis (granularity). The instrument computes a dynamic separation line between the granulocytes (orange) and the eosinophils (1a). Analysis-areas for haemozoin (Hz) containing monocytes: a) any purple dot above grey line (1a), b) any purple event above grey horizontal line (1b). Analysis area for Hz-containing granulocytes: area (gate) defined by area surrounded by solid black line (2). Analysis area for depolarizing blue coded events: area above solid black line (3). For detailed description on constructing the areas and cut-off lines, please see Methods section. Blue = lymphocytes, purple = monocytes, orange = granulocytes, green = eosinophils (or "mis-classified" Hz-containing granulocytes).

The Cell-Dyn^® ^instruments generate a five-part differential white blood cell count, using scatter flow cytometric principles based on the manufacturer's patented multi-angle-polarized-scatter-separation (M.A.P.S.S.^®^) [21] as described elsewhere [22,23]. The instrument aspirates 120 μL of peripheral blood. It than dilutes and gently lyses the red blood cells. A fixed volume (78 μL) of the final 1:51 dilution is than analysed. Given these values, it is possible to calculate the number of events that the instrument analyses to generate the FBC result. The events analysed correspond to the number of leukocytes/μl of blood multiplied by 1,529, with an upper limit of 10,000 events. For each event, data is acquired in one of 256 channels and these datasets are temporarily kept in a list mode file to generate the numeric FBC result and the graphic output that appears on the monitor. However, the list mode data is not accessible to the instrument's operator and is eventually deleted after the analysis. For the graphic output shown on the screen, only the first 5,000 of the total number of all gated events are utilised (Bodo Roemer, personal communication). The instrument has been shown to detect Hz-containing monocytes and granulocytes that are represented in a dot plot (granularity/lobularity) on-screen (Figure 1), as described elsewhere [11]. As the raw data files containing the list mode data were not available, screenshots were taken. The area of the granularity/lobularity plot was transformed into a bitmap image and analysed using ImageJ, a public domain image processing program [24]. Due to the custom screen resolution of the Cell-Dyn^® ^instruments and the transformation of the image, the maximum resolution of the analysed bitmap image was 140 × 140 pixels, rather than the 256 × 256 channels.

In the granularity/lobularity plot, two different areas were defined to classify monocytes as pigment-containing monocytes (PCM), shown in Figure 1: (i) any purple dot above a diagonal separation line, generated automatically by the instrument to distinguish between eosinophils and all other leukocytes, and (ii) any purple dot found above a horizontal line with 5 pixels distance from the x-axis. Concerning Hz-containing neutrophils (PCN) a different strategy had to be used, as the instrument "miss-classifies" Hz-containing neutrophils as eosinophils (both shown as green-coded events) [25]. Thus, a special area (gate) was created to identify PCN, with the intention to exclude eosinophils. In accordance with studies using flow cytometric cell sorting [13], the largest possible gate to the left and above the usual location of the eosinophil population was created that did not contain any eosinophils when this gate was applied to the FBC results from children without malaria (Figure 1, area defined by line 2). This gate was than applied to the dot plots in children with malaria and any green coded event within this area was considered to represent a PCN.

A higher degree of depolarization (higher y-axis value; granularity) may be caused by either a higher amount of phagozytosed Hz or larger Hz crystals. Thus, Hz-containing monocytes and granulocytes with higher depolarization values contain possibly more Hz and thus could be correlated with severity. To test this hypothesis, two "severity indices" with the intention to weigh the degree of depolarization were constructed: (i) the sum of the y-axis values of Hz-containing monocytes and granulocytes and (ii) the sum of the y-axis values after mathematical transformations (square-root and logarithm of the y-axis values). These indices were then used to determine a meaningful cut-off value that would allow to distinguish between severe and non-severe malaria.

During the analysis of the granularity/lobularity plot it was noted that blue coloured events, that usually represent lymphocytes, showed depolarization (Figure 1). A horizontal-line with 10 pixels distance from the baseline was used to determine the number of these events in the children with malaria.

Data was analysed using SPSS 14.0 software. The student t-test for unpaired samples with unequal variance was used to test quantitative data and the chi-square test to test qualitative data.

## Results

During the study period a total of 368 children (54% female, 46% male) were included, of which 152 had falciparum malaria and 216 children were either healthy or had other diseases. More than 88% of malaria occurred in children older than one year, while 99.1% of all children without malaria were below one year of age. The children in the malaria group were significantly older than the children without malaria (mean age: 3.7 years and 0.6 years, respectively; p < 0.05). Of the 152 children with malaria, 48 had severe malaria, classified as 15 cases with severe anaemia, 13 cases with hyperparasitaemia, three cases with hypoglycaemia and 17 children with cerebral malaria. In three cases, children had both cerebral malaria and severe anaemia, which were included in the cerebral malaria group. The mean age of children with severe malaria (3.8 years) and non-severe malaria (3.4 years) was not significantly different (p = 0.27).

The FBC results are shown in Table 1 and Table 2. Comparing children with and without malaria all FBC-parameters were significantly different, except for the eosinophil count (Table 1). The platelet count was significantly lower (p < 0.001) in the children with malaria as compared to those without malaria (Table 1) and, interestingly, the values showed very little overlap between both groups. When only looking at children with malaria, children with severe malaria had slightly higher mean WBC values than children with non-severe malaria. The differences reached statistical significance only for the total WBC count and the neutrophil count (Table 2). While haemoglobin levels were significantly lower in severe malaria than non-severe malaria, no difference was found for the platelet counts (Table 2).

**Table 1:** FBC results of children with and without malaria

**FBC-Parameter**	**no malaria (n = 216)**		**malaria (n = 152)**		**p-value**
	**mean**	**10^th^/90^th ^percentile**	**mean**	**10^th^/90^th ^percentile**	
**total WBC**	9.5	9.2/10.0	8.7	4.3/13.9	p < 0.05
**neutrophils**	2.7	1.1/4.7	3.8	1.7/6.6	p < 0.001
**lymphocytes**	5.5	3.3/8.0	3.0	1/6.9	p < 0.001
**monocytes**	0.8	0.2/1.4	1.3	0.3/2.6	p < 0.05
**eosinophils**	0.4	0.1/0.9	0.4	0/0.7	p = 0.63
**platelets**	428	286/594	137	54/243	p < 0.001
**haemoglobin**	9.8	8.5/11.0	7.8	5.2/10.5	p < 0.001

Values in thousand/μL, except haemoglobin in g/dL. Children with malaria were significantly older (mean: 3.7 years) that the ones without malaria (mean: 0.6 years) and the reference values for leukocytes and Hb are inherently different between the two age-groups (see discussion).

**Table 2:** FBC results for children with non-severe and severe malaria

**FBC-Parameter**	**non-severe malaria (n = 104)**		**severe malaria (n = 48)**		**p-value**
	**mean**	**10**^**th**^**/90**^**th**^**percentile**	**mean**	**10**^**th**^**/90**^**th**^**percentile**	
**total WBC**	8.1	4.2/13.0	10.0	4.8/18.2	p < 0.05
**neutrophils**	3.5	1.5/6.1	4.6	1.9/7.6	p < 0.05
**lymphocytes**	2.9	1.0/5.9	3.2	1.0/7.3	p = 0.41
**monocytes**	1.2	0.4/2.1	1.6	0.3/3.8	p = 0.09
**eosinophils**	0.3	0/0.5	0.3	0/0.9	p = 0.89
**platelets**	141	61/228	128	44/266	p = 0.41
**haemoglobin**	8.6	6.3/10.7	6.7	4.4/9.7	p < 0.001

Values in thousand/μL, except haemoglobin in g/dL.

**Figure 2:**
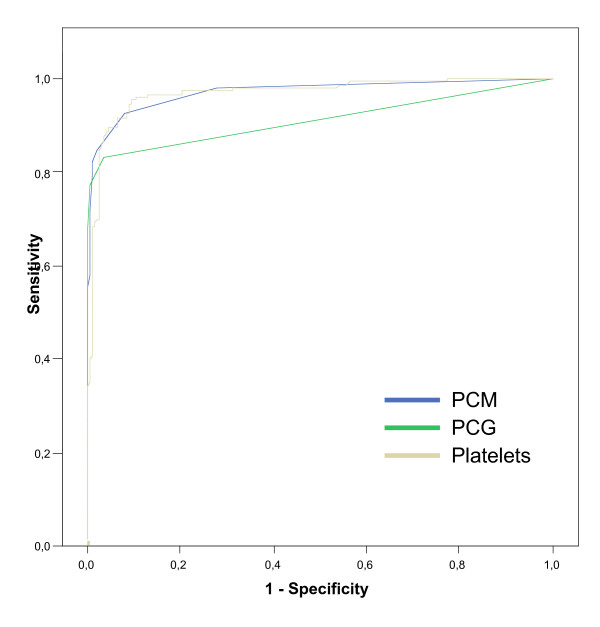
**ROC-curve: Accuracy of thrombocyte count, Hz-containing monocytes and granulocytes for malaria diagnosis**. The area under the curve indicates high accuracy for all three parameters: 0,97 for monocytes and platelet count and 0,91 for granulocytes (0,5–0,7: low accuracy, 0,7–0,9: moderate accuracy and >0,9 high accuracy).

Sixtyfive percent of children with malaria had < 150,000 thrombocytes/μL. Given the little overlap of the platelet counts between the malaria and non-malaria groups we computed a Receiver-Operator-Characteristic (ROC) curve (Figure 2). The ROC curve showed an area under the curve (AUC) of 0.97 which indicates a test with high accuracy (high accuracy = AUC > 0.9). Using a cut-off value of 250,000 thrombocytes/μL to predict malaria yielded a sensitivity of 92% and a specificity of 93%. However, employing the often-used 150,000 thrombocytes/μL cut-off gives values of 66% for sensitivity and 99% for specificity.

The other parameters that were analysed for their usefulness to diagnose malaria were the presence of Hz-containing leukocytes (PCM, PCN) detected by the Cell-Dyn 3000^® ^instrument. Concerning Hz-containing monocytes, no significant difference was observed between the two defining lines (Figure 1, lines 1a and 1b). Thus, in all subsequent analysis the horizontal line (1b) was used. The presence of ≥ 1 purple dot above this threshold line gave a sensitivity of 96% and a specificity of 96%. The detection of PCN, defined by the presence of green dots in the analysis area (Figure 1) yielded results for sensitivity and specificity of 85% and 96%, respectively. The ROC-curve analysis for both parameters showed that both tests have a high accuracy (Figure 2). When the detection of both cell types was combined (presence of PCM *or *PCN) the sensitivity was 97% and the specificity of 93%.

The detection of PCL by microscopy was compared with results from the Cell-Dyn^® ^instrument which showed that the analyser detected significantly more patients harbouring PCL (p < 0.01) (Table 3). This was not unexpected as the instrument analysed significantly higher numbers of monocytes and granulocytes when compared to microscopy (Table 3). Furthermore, it was observed that the microscopic identification of PCL in thick films was complicated due to the morphologic alterations caused by the staining process, and that it was often difficult to distinguish unambiguously between granulocytes and monocytes.

**Table 3:** Comparison of microscopic and automated detection of Hz-containing leukocytes

	**non-severe malaria (n = 104)**	severe malaria* (n = 48)	**severe anaemia (n = 15)**	**cerebral malaria (n = 17)**
**Patients with PCM**				
Microscopy	91.3%	100%	100%	100%
Cell-Dyn 3000^®^	94.3%	100%	100%	100%
**P**	NS	NS	NS	NS
**Patients with PCN**				
Microscopy	56,7%	91,7%	80,0%	100%
Cell-Dyn 3000^®^	80,7%	96,8%	86,7%	100%
P	p < 0,0001	NS	NS	NS

Microscopy: Observation of 200 granulocytes and 100 monocytes in Giemsa-stained thick film. The Cell-Dyn 3000^® ^analysed a mean of 4,175 granulocytes (range: 800–8,830) and a mean of 1,364 monocytes (range: 230–3,660).

* Severe malaria includes the following cases: severe malaria (n = 15) and cerebral malaria (n = 17), as shown in two right colums; hyperparasitaemia (n = 13), hypoglycaemia (n = 3).

P = chi square test, NS: not significant (>0,05)

**Figure 3:**
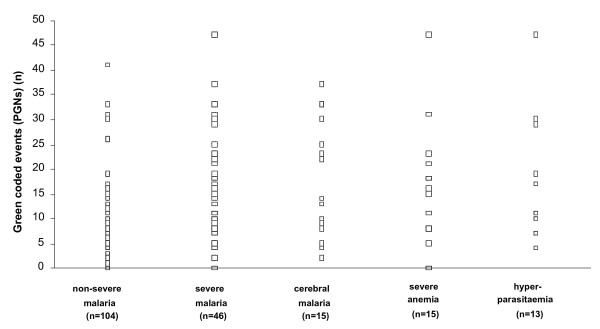
**Automated detection of Hz-containing leukocytes and disease severity**. Scatterplots of (a) purple coded events (PCM, Hz-containing monocytes) and (b) green coded events (PCN, Hz-containing granulocytes) distribution in children with non-severe and severe malaria (left two colums), and in three subgroups of severe malaria (severe anaemia, cerebral malaria and hyperparasitaemia, right three colums). Solid bar represents mean, dashed bars 95% confidence interval about the mean for the standard error. See text for outliers that were removed.

The results for the automated detection of Hz-containing leukocytes in severe and non-severe malaria are shown in Figure 3. Considering the PCM, four datasets were considered to represent outliers (cases with >100 PCM) and were not considered for statistical analysis: one case each of cerebral malaria (185 PCM), severe anaemia (169 PCM), hypoglycaemia (127 PCM) and one case of non-severe malaria (109 PCM). Correspondingly, two datasets of Hz-containing granulocytes were also considered to have outliers (cases with > 50 PCN): both were children with cerebral malaria (66 PCN and 75 PCN). The higher number of Hz-containing monocytes in severe malaria (mean: 25.4) as compared to non-severe malaria (mean: 19.3) did not reach statistical significance (p = 0.14). In the subgroups, the group with severe anaemia (mean: 39.9) appeared to have higher numbers of PCM as compared to the children with cerebral malaria (mean: 23.3), or hyperparasitaemia (mean: 14.8). In contrast to this, the difference in Hz-containing granulocytes was significantly different (p < 0.0001) between the severe (mean: 15.7) and non-severe malaria (mean: 7.5) groups. However, the difference appeared to be marginally bigger for the children with cerebral malaria (mean: 18.0) than for the severe anaemia (mean: 15.3) and hyperparasitaemia groups (mean:16.3).

The number of Hz-containing leukocytes showed a wide distribution with an overlap between the non-severe and severe malaria groups (Figure 3). This did not allow for calculating a meaningful cut-off value to distinguish between both groups. Computing of the respective ROC curves showed an AUC of 0.62 for PCM and of 0.75 for PCN, indicating a test with low accuracy. When attempting to include the degree of depolarization, by calculating the severity indices, it was found that these indices did not give better results than the total number of the respective Hz-containing leukocytes to distinguish between severe and non-severe malaria.

Concerning the presence of depolarizing blue coloured events (lymphocytes), they were found in 77 of 152 children with malaria (51%). The mean number of these events in the 77 children was 4.2 (range: 3 – 24). Although they appeared to be evenly distributed between the non-severe and severe malaria groups, they seemed to be more frequent in children with anaemia, with 11/14 FBC results yielding depolarizing blue coloured events.

## Discussion

This study underpins the value of thrombocytopenia for malaria in the investigated age groups and in the setting of a Central African rainforest area and provides more insight into the usefulness of PCL analysis as a prognostic marker in malaria. It furthers the knowledge about the utilization of scatter flow cytometry for this purpose, which may be applied in future in low-cost, robust devices needed for other applications such as CD4+ cell identification.

Concerning the FBC parameters, most of the values showed a significant difference between the malaria and non-malaria groups. As the children with malaria were significantly older (mean: 3.7 years) than the ones who had no malaria (mean: 0.6 years), these results may in great part simply reflect the different reference values in both age groups [26]. This effect may even be more pronounced in this study as children below one year and especially below three months tend to have rather higher WBC counts and higher lymphocytes and monocytes than older children [26]. Although, an age-dependent difference in Hb reference values exists, lower values are observed in younger children [26]. Thus, the observed lower Hb levels in the older children with malaria are even more relevant. Not unexpectedly, the Hb levels were lowest in children with severe malaria, followed by uncomplicated malaria and highest in the non-malarious group (Table 1 and Table 2).

A notable observation was not only the significantly different platelet count between the malaria and non-malaria groups, but even more so, that almost no overlap of values was observed. However, it could be argued again that this observation reflects rather the different age in both groups. Interestingly, most textbooks give only one set of reference values for all age groups, which implies the same or very similiar values for children of different ages [26]. On the other hand, studies that addressed the reference values in African children found an age-dependent difference [25]. However, it appears hat the lower value appears to be constant (around 150,000), while only the upper value is much higher in younger children. Consequently, the interval between lower and upper value is much wider in younger children [27].

Given the frequently-used cut-off value of 150,000 platelets/μL, this cut-off would have identified 66% of malarious children, while almost none of the children without malaria had such a value (specificity 99%). These values are in keeping with other reports and confirm the usefulness of a platelet count as marker of acute malaria. In fact, ROC curve analysis indicates that this parameter showed a high degree of accuracy (Figure 2); and even using a rather high cut-off value of 250,000 platelets to distinguish malaria from non-malaria cases gave good values for sensitivity (92%) and specificity (93%). Nonetheless, this finding has to be confirmed in other regions and comparing different age groups. Interestingly, when the platelet counts were compared in the malaria group, no further difference was observed between uncomplicated and severe malaria and no association could be established with the type of severe malaria. However, despite an involvement in the pathophysiological process of malaria, a recent study showed that malaria patients had significantly more platelet aggregates than individuals without malaria [28]. It may be possible that some degree of thrombocytopenia may not be disease-related, but rather being caused by erroneous platelet counts due to plalelet aggregation.

The study did also confirm that the CD 3000^® ^instrument has a large potential for the rapid and accurate detection and enumeration of PCL as it identified more PCL-positive samples than microscopy (Table 3). This became particularly apparent for samples containing PCN. In fact, the microscopic enumeration of Hz-containing WBC in Giemsa-stained thin or thick films can be difficult. In thick films, the distorted cell morphology can make it very difficult to reliably identify a granulocyte or monocyte. Furthermore, the stain may leave artefacts that are sometimes difficult to discern, and small Hz crystals may be easily overlooked. Polarizing or dark field microscopy are easy and inexpensive methods. Although they have been shown to be helpful [29,30], so far none of the studies on PCL seems to have employed these techniques (Table 4) [7-9,31,32]. On the other hand, detecting PCL on thin films is very time consuming, given the number of cells that have to be screened. Furthermore, previous studies did observe rather limited numbers of monocytes and granulocytes to identify Hz-containing cells. This translates into a significant imprecision of the results (Table 4), a fact well known for the manual 100-cell FBC diffential counts [3]. Contrary to this, the CD3000^® ^did analyse hundreds, often thousands of monocytes and granulocytes (Table 3) with high precision.

**Table 4:** Selected studies investigating Hz-containing leukocytes and disease severity

**Reference year published**	**Mujuzi et al. 2006 **[32]	**Lyke et al. 2003 **[9]	**Amodu et al. 1998 **[8]	**Phu et al. 1995 **[31]	**Metzger et al. 1995 **[7]
**place of study/endemicity**	Northern Uganda intense holoendemic	Mali/intense seasonal	Nigeria/holoendemic	Viet Nam/ND	Gabon/hyperendemic
**population**	children, 6–59 months old	children, 3 months to 14 years old	children	adults	children, 6 months to 14 years old
**study size**	(n = 208), severe (n = 99)/uncomplicated (n = 99)	(n = 516), severe (n = 163), uncomplicated (n = 163), healthy (n = 164)	(n = 146), cerebral (n = 43), uncomplicated (n = 43), asymptomatic (n = 32), no malaria (n = 28)	(n = 300), cerebral (n = 168), fatal (n = 40)	(n = 73), severe (n = 42), mild (n = 31)
**WBC count method**	assuming 8,000/μL	assuming 7,500/μL	assuming 8,000/μL	assuming 8,000/μL	ND
**Hz-leucocyte counting method**	thin film/counting 500 leukocytes	thin films/counting 30 monocytes, 100 PCN	thick films/counting 30 monocytes, 100 PCN	thin & thick films/counting 30 monocytes, 100 PCN	thick films/counting 100 monocytes, 100 PCN
**quality assessment of microscopy**	ND	10% of slides re-examined by 2nd micoscopist	ND	3 slides observed by 10 microscopists/1 slide examined 10× times by the same microscopist	count independently by two investigators
**Result of quality assessment**	ND	Kappa coefficient for: – PMN: 0,88 – mono: 0,77	ND	-mono: 125, 76 and 40	ND

ND: not determined/done or not given

The detection of PCL was a very accurate marker of malaria, with results for sensitivity and specificity > 90%, which is higher than results from previous studies from non-endemic as well as endemic regions [11,13-15,25,33-36]. One explanation may be the fact that in contrast to previous studies only children were included. However, PCN were an inferior marker of malaria than were PCM. Possibly, granulocytes are less frequent because (i) they may only be recruited in more severe disease, that is, when there is more haemozoin in circulation [37]; and (ii) they have a shorter half life in the circulation than monocytes [38]. On the other hand, it cannot be excluded that the instrument detected more PCN that were lying outside the created gate and were thus "wrongly" considered to be "true" eosinophils and consequently excluded from analysis.

When analysing PCL and disease severity, several aspects became apparent. The number of PCM was different between the severe and non-severe malaria groups; however, in contrast to several previous studies [8,32,39] this did not reach statistical significance. Yet, PCM seemed to be highest in the severe anaemia group as reported before [9], a finding that is in line with previous observations that Hz may be involved in the pathogenesis of anaemia [40]. On the other hand, PCN were significantly more frequent in severe malaria, with highest values in cerebral malaria. Interestingly, one study reported that granulocytes were recruited after Hz administration in a mouse model in a dose-dependent manner [37]. Possibly, in severe malaria more Hz is present in the circulation and thus more granulocytes are actively ingesting Hz, which would also explain why PCN are an inferior marker for malaria as their numbers in non-severe malaria may be much lower than PCM. Although some of these observations have been described before in studies using microscopy, it is of note that the results between these studies vary immensely. For example, the percentage of patients with severe malaria that had PCM varied between 65% and 85%, and those that had PCN varied between 37% and 85% (Table 5). Although these discrepancies could be due to different populations studied, it seems equally likely hat they reflect the difficulty in obtaining reliable results by light microscopy. Furthermore, some studies did calculate the total amount of HZ-containing leukocytes based on an assumed count of 7,500 or 8,000 WBC per microliter [8,9,38,39]. In this study, the mean WBC count between severe and non-severe malaria differed by more than 2,000/μL (Table 2), and thus results for absolute counts are bound to be very different if the "real" WBC count would have been used.

**Table 5:** Comparison of results of some studies on Hz-containing leukocytes

**Reference Year published**	**Mujuzi et al. 2006 **[32]	**Lyke et al. 2003 **[9]	**Amodu et al. 1998 **[8]	**Metzger et al. 1995 **[7]
**Patients with PCM (%):**				
**Severe Malaria**	68	85	100	100
**Uncomplicated Malaria**	37	70	95	87
**Healthy Control**	ND	6	96	ND
**Patients with PCN (%):**				
**Severe Malaria**	37	86	100	95
**Uncomplicated Malaria**	1	54	95	32
**Controls**	ND	2	71	ND
**Monocytes containing Hz (%) = PCM**	ND	mean	median (IQR)	median (range)
**Severe Malaria**		14	53 (37–70)	24 (2–57)
**Uncomplicated Malaria**		5	17 (13–30)	7 (0–45)
**Healthy Control**		0,03	29 (20–35)	ND
**Neutrophils containing Hz (%) = PCN**	ND	mean	median (IQR)	median (range)
**Severe Malaria**		4	27 (15–38)	2 (0–15)
**Uncomplicated Malaria**		2	9 (4–17)	0 (0–7)
**Healthy Control**		0,03	2 (0–6)	ND
**total number of PCM (μL)**	median (range)	mean (range)	ND	ND
**Severe Malaria**	32 (0–640)	216 (0–3,420)		
**Uncomplicated Malaria**	0 (0–272)	94 (0–1,698)		
**Healthy Control**	ND	4.9 (0–285)		
**total number of PCN (μL)**	median	mean (range)	ND	ND
**Severe Malaria**	(range)	349 (0–3,721)		
**Uncomplicated Malaria**	0 (0–80)	64 (0–534)		
**Healthy Control**	0 (0–16) ND	1 (0–81)		

ND: not done or not given

This study has several limitations. First, the age between the malaria and non-malarious children was significantly different, thus making comparisons for many FBC parameters difficult. Furthermore, it seems likely that access to the original raw data from the CD3000^®^, in list mode format, would have shown that many more PCL were detected, but were either not shown on-screen (because of the 5,000 cells limit), or were lost during the "screen shot" transformation that resulted in a decrease from 256 × 256 channels to a 140 × 140 pixel image. In fact, the number of coloured pixels that represent leukocytes were analysed and compared to the total number of leukocytes in a subset of FBC results. It was found that the granularity/lobularity plot (Figure 1) contains on average only some 400–600 coloured pixels, while in the corresponding samples 7,000–10,000 events were analysed. Consequently, the original list mode data contains up to 10 times more analysed events than those analysable by the investigators. Even considering that only 5,000 events are used to generate the graphic screen-output, the factor for loss of information is still in the order of five times. Therefore, it would be desirable to confirm the present findings by counter-checking the CD3000^® ^results using flow cytometry and marking the leukocytes with anti-CD14 and anti-CD16 antibodies.

An interesting finding is that there are lymphocyte-like, blue coloured (in the Cell Dyn^® ^technique's way) events that are highly depolarising. However, lymphocytes do not phagozytose and consequently, these cells cannot be lymphocytes, but must be phagocytic cells with cell characteristics that are similar to lymphocytes (rather small cells, with a high nuclear to cytoplasm ratio and a rather round nucleus, i.e. either NK cells or peripheral blood phagocytic cells. Those cells should be analysed by flow cytometry to establish their nature and possible role in the pathophysiology of malaria.

## Conclusion

In the age group examined in the Lambaréné area, platelets are an excellent adjuvant tool to diagnose malaria. Pigment-containing leukocytes (PCL) are more readily detected by automated laser-flowcytometry than by microscopy. Mechanical Hz detection by an instrument as used here is a reliable diagnostic tool and correlates with disease severity. However, clinical usefulness as a prognostic tool is limited due to an overlap of PCL numbers recorded in severe versus non-severe malaria; possibly because of a detection algorithm not geared towards this task, and data lost during processing. Newly described 'lymphocyte-like' gated events warrant further examination and should be analysed by flow cytometry to establish their nature and role in the pathophysiology in malaria.

## Abbreviations

FBC: full blood count; Hb: haemoglobin; Hz: haemozoin or malaria pigment; IPTi-SP: Intermittent Preventive Treatment in infants of malaria with sulfadoxine-pyrimethamine; PCL: (malaria)pigment-containing leukocyte(s); PCM: (malaria)pigment-containing monocyte(s); PCN: (malaria)pigment-containing neutrophil(s) or granulocytes; WBC: white blood cell count

## Authors' contributions

TH and MPG designed the study, collected and analysed data and prepared the manuscript.

ML helped designing the study, collected and analysed data and contributed to the manuscript's final version.

MP and SO helped collecting the data and contributed to the manuscript's final version.

BL contributed to study design, data analysis and the manuscript's final version.

PGK contributed to the study design and to the manuscript's final version.
